# A reconfigurable visual-programming library for real-time closed-loop cellular electrophysiology

**DOI:** 10.3389/fninf.2015.00017

**Published:** 2015-06-23

**Authors:** István Biró, Michele Giugliano

**Affiliations:** ^1^Theoretical Neurobiology and Neuroengineering, University of AntwerpAntwerpen, Belgium; ^2^Department of Computer Science, University of SheffieldSheffield, UK; ^3^Laboratory for Neural Microcircuitry, Brain Mind Institute, École Polytechnique Fédérale de LausanneLausanne, Switzerland

**Keywords:** electrophysiology, experimental control, cellular neurobiology, dynamic clamp, closed loop, active electrode compensation, extracellular stimulation, response clamp

## Abstract

Most of the software platforms for cellular electrophysiology are limited in terms of flexibility, hardware support, ease of use, or re-configuration and adaptation for non-expert users. Moreover, advanced experimental protocols requiring real-time closed-loop operation to investigate excitability, plasticity, dynamics, are largely inaccessible to users without moderate to substantial computer proficiency. Here we present an approach based on MATLAB/Simulink, exploiting the benefits of LEGO-like visual programming and configuration, combined to a small, but easily extendible library of functional software components. We provide and validate several examples, implementing conventional and more sophisticated experimental protocols such as dynamic-clamp or the combined use of intracellular and extracellular methods, involving closed-loop real-time control. The functionality of each of these examples is demonstrated with relevant experiments. These can be used as a starting point to create and support a larger variety of electrophysiological tools and methods, hopefully extending the range of default techniques and protocols currently employed in experimental labs across the world.

## Introduction

The application of Control Theory is increasingly gaining popularity in Neuroscience and Neuroengineering (Potter et al., 2014). Based on the well-defined, precisely timed and automatically regulated activation of neurons by an external stimulus, several new paradigms and therapeutic strategies have been proposed. In terms of preclinical applications, closed-loop deep brain electrical stimulation is an example of this trend and has been shown to be superior to conventional, i.e., open-loop, operation. In fact, regulating, in real-time, the temporal pattern and intensity of extracellular electrical repeated stimuli as a function of the actual neuronal activity, is proven to reduce the adverse effects of habituation (Carron et al., 2013). This not only improves efficacy of stimulation but also boosts power efficiency of the implanted devices. These promising results have then stimulated further for the exploration of new treatments for epilepsy (Berenyi et al., 2012; Beverlin Ii and Netoff, 2013; Paz et al., 2013) and Parkinson's disease (Feng et al., 2007; Rosin et al., 2011; Gorzelic et al., 2013; Priori et al., 2013). Notably, in the context of advanced neuroprosthetics, closed-loop real-time strategies have also been explored as a proof of concept for function recovery, in an animal model of brain damage (Guggenmos et al., 2013), and very recently enabled a realistic bidirectional sensori-motor operation in amputees (Raspopovic et al., 2014).

Beyond its therapeutic applications and perspectives, closed-loop experimental paradigms have been widely considered for addressing fundamental research questions in Neuroscience *in vitro* and *in vivo* (Arsiero et al., 2007). For instance, progress has been reported in artificially-assisted rewiring of microcircuits (Bonifazi et al., 2013), in reverse-engineering the time-scales underlying neural and synaptic responses (Wallach, 2013; Reinartz et al., 2014), in studying plasticity (Franke et al., 2012), in offering a theoretical ground for steering coherence of neuronal oscillations (Witt et al., 2013), in recreating realistic *in vivo-like* activity regimes in *in vitro* preparations (Destexhe et al., 2003; Bal and Destexhe, 2009), or in exploring the impact of re-engineered or synthetic cellular and synaptic components (Robinson and Kawai, 1993; Sharp et al., 1993; Economo et al., 2010). Particularly for basic research, the ease of access to community-contributed tools for real-time closed-loop experiments is imperative and is one of the priorities of Neuroinformatics.

Many excellent general-purpose software packages, as well as *ad hoc* hardware/software solutions, have already been presented (Benda et al., 2007; Muñiz et al., 2009; Lin et al., 2010; Rolston et al., 2010; Zrenner et al., 2010; Chamorro et al., 2012; Newman et al., 2013; Linaro et al., 2014), ultimately offering an upgrade of the conventional techniques and protocols routinely used for cellular electrophysiology across laboratories worldwide. However, most of the community-contributed packages are rather specialized or require moderate to substantial computer proficiency to be installed and operated, as well as some degree of fluency in procedural computer programming to be customized or scripted.

Here we present our contribution to the community, in the form of a reconfigurable visual-programming library of software components, entirely based on MATLAB and Simulink (https://bitbucket.org/mgiugliano/pc_neuron_simulink), and currently in use in our experimental laboratory. While MATLAB is a high-level programming language, widely spread in scientific and technical computing worldwide with support for signal and image acquisition/processing, symbolic math, control systems, and computational biology, Simulink is entirely graphical environment. In fact, as an add-on to MATLAB, Simulink features a graphical block diagramming tool and a set of block libraries, integrating seamlessly with MATLAB and widely adopted for automatic control, digital signal processing, multidomain simulation, and Model-Based Design.

Such a library contains the basic blocks necessary for building a range of popular open and closed-loop experiments in cellular electrophysiology. Inspired by the many efforts to improve user interactions with the computer and ultimately its friendly programming, we explored the benefit of a visual programming language readily available as a toolbox of a numerical computing platform that is already very popular and widespread in academia. Our library combines modularity and customizability with the existing wide range of natively supported hardware for data acquisition. It offers users with little knowledge in programming the possibility to develop experimental protocols by intuitively reusing, manipulating and arranging elements and subcomponents graphically rather than textually. As in electrophysiological equipment, where cables and connectors physically connect hardware units, the availability of elementary primitives in our library enables the implementation of e.g., standard voltage or current-clamp intracellular stimulation and recording protocols upon visual design of the software schemes, or “models.” However, more advanced applications also are within immediate reach, including the active electrode compensation (Brette et al., 2008), conductance-injection (Bal and Destexhe, 2009), neuronal or synaptic response probability clamping (Wallach and Marom, 2012; Reinartz et al., 2014) and spike-frequency clamp (Miranda-Dominguez et al., 2010; Linaro et al., 2014). The library and the examples discussed in this contribution will enable users, who are not expert programmers and who share a limited computer proficiency, an alternative approach to the design and customization of experimental protocols and applications requiring the execution of real-time feedback loop tasks.

## Materials and methods

### General description

**Figure 1:**
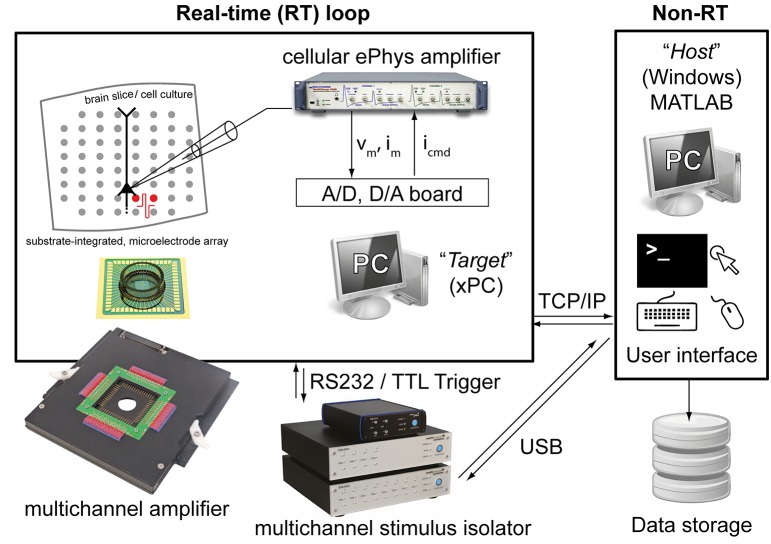
**The experimental setup employed for validating our Library**. Two personal computers (PCs) are connected together: one (i.e., the host) runs Windows and the other (i.e., the target) runs the xPC operating system. While the host allows user interaction, system reconfiguration by visual programming, data storage and control, the target operates independently and in real-time and it is programmed remotely by the host. The target is also equipped with a standard data acquisition board and with a serial port, connected to a patch-clamp amplifier and/or an extracellular stimulus isolator.

The Library proposed here is fully based on MATLAB and Simulink (The Mathworks, Natick, MA, USA) and additionally takes advantage of the xPC MATLAB Toolbox for real-time operation. The last is an environment for simulating and testing Simulink models in real-time on a physical hardware system. Therefore, two personal computers (PCs) are required (Figure 1): (i) the *target PC* running the xPC real-time operating system and equipped with a CD drive, data acquisition (DAQ) hardware, and an Ethernet network card; (ii) the *host PC* running MATLAB on any operating system (e.g., Windows 7) and equipped with keyboard, mouse, screen, a CD-burner and an Ethernet network card. These two PCs are directly connected to each other via a dedicated “cross-over” Ethernet cable (i.e., using TCP/IP, UDP or serial communication protocols), and interfaced to existing electrophysiological equipment as sketched in Figure 1.

**Figure 2:**
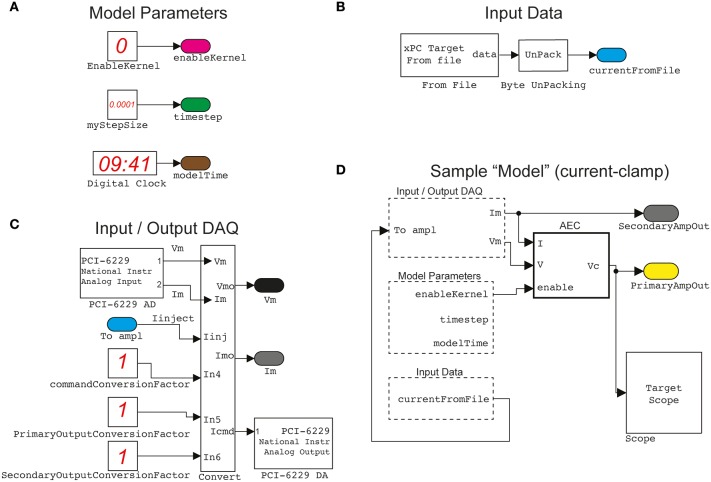
**A sample of elementary building blocks and their visual configuration**. Basic parameters, such as the sampling interval and a switch enabling the operation of the Active Electrode Compensation (as a stand-alone block) are encapsulated **(A)** in a block named ModelParameters. Another block, named InputData **(B)** provides an interface to arbitrary stimulation waveforms (e.g., time-varying current commands, voltage commands, conductance commands, etc.), generated off-line by the host PC, and connected to the Input/OutputDAQ block. This operates the digital-to-analog and analog-to-digital conversion **(C)** via the DAQ card and incorporates low-level hardware configurations. Together with the other two blocks, as well as standard Simulink components (e.g., the Scope for signal monitoring), the Input/OutputDAQ can be employed to graphically configure **(D)** a sample (i.e., current-clamp) protocol. The actual Simulink blocks configuration diagrams, displayed in the documentation of our Library, have been adapted and simplified for clarity.

The *target* PC does not require any operating system and specific software installation: it boots from a special “live” disk, configured using the host computer and burned once for all on a CD-ROM. This step requires executing, on the *host* PC, a custom-made setup script provided with our Library, or following the step-by-step procedure outlined in the official xPC documentation. Once done, the *target* PC boots from the CD-ROM for each use and becomes fully controlled by the *host* computer, which exclusively employs MATLAB/Simulink to load and customize the experimental protocol. In the details, sample configurations schemes are provided with our Library (i.e., in the following referred to as *graphical Simulink models* or simply as *models*) and can be instantiated, reconfigured, or combined together as building blocks, while internally incorporating standard Simulink components or custom-made MATLAB/C/C++ routines. Once a model has been visually arranged and defined (e.g., Figure 2), the *host* PC compiles and uploads its code to the *target* PC that executes it in real-time. Parameters initialization and model execution control are performed on the *host* PC, always through MATLAB.

As the *target* PC executes the tasks of the model in a real-time loop, communication with the *host* PC occurs asynchronously and non-real-time, via TCP/IP or UDP protocols. The *host* can then receive inputs and data from the *target* and the electrophysiological equipment connected to it, by an automated additional *receiver* Simulink model, running on the *host* and provided in our Library. This takes care, among other things, of data storage on the hard drive of the *host*. Alternatively or additionally, simple MATLAB instructions can be used to perform additional analysis or even to alter the target model parameters, during its execution. In its present form, our Library thus restricts the operations of the *host* computer largely to monitoring and control of the *target*, encouraging the use of its largely unused computing resources for more demanding, non-real-time processing (e.g., pre-processing, data-analysis and visualization of raw or processed data).

Besides the run-time data-transfer from the *target* to the *host*, several other methods of data streaming and acquisition are also supported under xPC. For example, by equipping the *target* PC with a large hard disk, data can be logged and stored locally and transferred to the *host* only at the completion of the entire task. More elaborate forms of customized data acquisition and transfer, such as triggered data logging, have also been implemented and demonstrated in the Results.

In the applications demonstrated in **Figures 8, 9**, the *host* PC has also been employed to interface via USB additional equipment, such as an extracellular electrical stimulus isolator. As in those applications, on the fly but not real-time operation was required, the *target* automatically instructs the *host* to re-program the isolator. In a similar circumstance, the components provided in our Library make the *target* instruct the *host* to initiate certain commands, allowing an ultimate interfacing with slower devices.

### Computer hardware

The *target* computer used in our test and validation was a modern PC based on the Intel i5-3330 3 GHz Quad Core Processor (Intel Corp., Santa Clara, California, United States), mounted on a BCM BC77Q Motherboard (BCM Advanced Research, Irvine, California, United States), equipped with 4 Gigabyte DDR3 PC12800 RAM memory. The *target* also mounted an IDE CD-ROM drive and a hard disk, which was formatted to include a 10 Gigabyte (FAT32) partition required by xPC for local data storage. In order to achieve higher performance of real-time operation, by experience we discovered that all USB ports on the Motherboard, but not serial ports, had to be disabled by directly operating on the BIOS configuration menus of the *target* PC. Therefore, any model requiring the control of an USB-operated device, such as the extracellular stimulus isolator that we tested (STG 2008, Multichannel Systems GmbH, Reutlingen, Germany) performed much more efficiently when connected to the *host* PC while using the TCP/IP communication to allow the *target* to instruct the *host* to interact with the isolator (**Figures 7, 8**). In this case, electrical extracellular stimuli had to be repeatedly delivered while off-line updating their DC intensity as a function of the neuronal response. The *host* PC was then instructed by the *target* to synthesize the needed stimulus waveform, upload it into the isolator via USB, and prepare the isolator to wait for an external TTL trigger signal (Figure 1). The real-time aspect of this operation is then represented by the repeated periodic generation of the trigger signal by the *target* PC, at a precise time.

One hundred Mbit/s network cards, connected directly via a “cross-over” Ethernet cable, provided sufficient data transfer speed for our specific purposes, via a TCP/IP connection between the *target* and the *host* PCs.

The *target* PC also contained a low-cost DAQ hardware board (PCI-6229, National Instruments, Zaventem, Belgium), which is supported by xPC and the default Simulink libraries. This offered complete 16 bits digital-to-analog and analog-to-digital signal conversions, interfacing and controlling a modern patch-clamp electronic amplifier (Axon Multiclamp 700B Microelectrode Amplifier, Molecular Devices, Sunnyvale, CA, USA), as well as digitally triggering external equipment such as the stimulus isolator mentioned above.

The *host* PC used in our test is a modern PC based on an Intel E8600 dual-core processor and equipped with 4 GB RAM. It runs Microsoft Windows 7 as the operating system and operates MATLAB/Simulink version 8.2.0.701 (R2013b).

### Brain tissue slices preparation

Tissue preparation was performed as described earlier (Köndgen et al., 2008) and in compliance with the guidelines of the Ethics Committee of the Department of Biomedical Sciences of the University of Antwerp. Briefly, 14–21 days old Wistar rats were anaesthetized with Isoflurane (IsoFlo, Abbott, USA), decapitated, and their brains quickly excised. 300 μm thick neocortical slices (parasagittal) of the somatosensory cortex were cut by a vibratome (VT1000 S, Leica Microsystems, Diegem, Belgium) in ice-cold artificial cerebrospinal fluid (ACSF). The ACSF contained (in mM) 125 NaCl, 25 NaHCO_3_, 2.5 KCl, 1.25 NaH_2_PO_4_, 2 CaCl_2_, 1 MgCl_2_, 25 glucose, balanced by 95% O_2_, 5% CO_2_ and adjusted to pH 7.3. The same solution was also employed after cut, to incubate slices at 36°C for at least 45 min, during the slice storage at room temperature, as well as during the electrophysiological recordings, performed at 32 ± 1°C. All chemicals were obtained from Sigma–Aldrich (Diegem, Belgium).

### Substrate-integrated microelectrode arrays (MEAs)

Brain tissue slices were laterally trimmed, to a width of ~5–6 mm, and coupled to glass-substrate arrays of 3D tip-shaped Pt microelectrodes (MEAs; 8 × 8 layout, 200 μm inter-electrode distance; Qwane Biosciences SA, Lausanne, Switzerland). The inner MEA area was previously coated with cellulose nitrate (Protran, Fisher Scientific, Belgium; 0.14 mg/ml in 100% Methanol) and replaced the bottom of the chamber of an upright microscope (Figure 1). Here, MEAs have been employed, in combination to patch-clamp intracellular recordings, to deliver multi-site extracellular stimulation by a stimulus isolator that generated current-controlled biphasic pulses (symmetric, positive phase first, lasting 200 μs—Figure 1). Stimuli were delivered in bipolar configuration via pairs of neighboring MEA microelectrodes, located in L1-L2/3 or in L5-6, not farther than 1.1 mm from the vertical axis of the targeted pyramidal cell (see the next subsection). Extending our current Simulink library to incorporate MEA recording equipment, and not only MEA stimulation, is possible by incorporating in our Library the Simulink blocks developed and distributed by Marom and collaborators (Zrenner et al., 2010).

### Intracellular recordings and stimulation

Patch-clamp recordings were obtained in the whole-cell configuration, under continuous perfusion (i.e., at a rate of 1 mL/min), from the soma of large pyramidal neurons of the cortical layer 5 (L5), under infrared differential interference contrast video-microscopy (DIC), using an upright microscope with 40× Olympus water-submersion objective (Slicescope, Scientifica, Uckfield, UK). As electrodes we employed filamented borosilicate glass pipettes (World Precision Instruments, Hitchin, UK), pulled by a horizontal puller (P-97, Sutter Instruments, Novato, CA, USA) to a resistance of 5-14 mW, when filled with intracellular solution (ICSF). ICSF contained (in mM) 115 K-gluconate, 20 KCl, 10 4-(2-hydroxyethyl)-1-piperazineethanesulfonic acid (HEPES), 4-adenosine triphosphate-Mg, and 0.3 Na_2_-guanosine triphosphate, 10 Na_2_-phosphocreatine, adjusted to a pH of 7.3.

The recording of the neuron membrane potential and the intracellular injection of current waveforms were performed by means of an Axon Multiclamp amplifier. Data was sampled at a frequency of 15 kHz and digitized at 16 bits/sample under the *target* PC, running the xPC operating system. Electrode resistance and capacitance compensation circuits offered by the amplifier were not used. Instead the Active Electrode Compensation (AEC) technique was employed in real-time, digitally compensating for the artifacts following current injection through the pipette (Brette et al., 2008) (see the block AEC in Figure 2D).

## Results

The Library described in our contribution is based and integrated in the existing object-oriented visual environment provided by MATLAB/Simulink. This constitutes a rather intuitive programming framework, where the solution of a complex task is approached at the functional instead of procedural/programmatic level. Starting from a set of function (virtual) blocks, available in the form of default or external libraries, their visual assembly through a graphical user interface upon drag-and-drop is performed by the user. While no knowledge of the inner working of each block is required, connections among the blocks and block free parameters must be specified, resulting in a cascade of processing steps laid out in space, instead of in time. The blocks available from default libraries, or those provided by our Library, are then designed to solve well-defined reduced tasks, ranging e.g., from a simple algebraic addition of signals (superposing two injected current waveforms, in current-clamp) to temporal filtering (smoothing the instantaneous firing rate and providing its temporal average), and from signal generation (realization of a noisy stochastic waveform) to more complex computations (convolving the total injected current with the estimated kernel of the patch-electrode). Simplicity, modularity, and reusability are therefore major advantages of this approach, which does not exclude a programmatic lower-level inclusion of MATLAB/C code for integration with earlier code or for further extension.

While the resulting combination of connected blocks, performing a given high-level task, is usually employed off-line to process data traces or to perform computer simulations, it can also be used in real-time and on-line. This is made possible by the availability of the Simulink real-time target (xPC) that allows real-time execution of the user-assembled blocks cascade, fed by or producing data through A/D D/A boards (e.g., connected to an amplifier). This allows to some extent the precise timing of execution, which is extremely important in real-time experimental protocols such as dynamic-clamp, without the need for developing *ad hoc* acquisition software.

Benefiting from advantages and simplicity, we offer a basic set of custom written blocks and illustrate their use as descriptions of use-case scenarios to ultimately demonstrate (i) the simplicity of replicating (less) conventional experimental protocols in cellular electrophysiology, and (ii) suggesting reusability of existing blocks for other applications. We also demonstrate how basic models can be extended for novel functionality and how new blocks are added. The following sections describe these concepts in more detail.

### Custom scripts, simulink blocks, and packages

By this methods paper, we aim at disseminating a reconfigurable visual-programming library, based on MATLAB/Simulink, developed and employed in our laboratory for routine experiments in cellular electrophysiology. A set of custom building blocks, MATLAB functions, Simulink “models” and documentation files will be made available from the website of the Authors as well as from the International Neuroinformatics Coordinating Facility software repository. This material has been prepared to facilitate the installation and use of our Library and to enable the user to rapidly replicate several experimental protocols, involving open- and closed-loop operation.

#### Available blocks

A list of blocks included in our library is given in Table 1, along with a short description of their functionality. More information is available from the user documentation and from the block-help within Simulink. These blocks can be used in combination with all other Simulink blocks, and serve as a basic set of elements, aimed for use in cellular electrophysiology.

**Table 1:** **Blocks provided by our library**.

**Block name**	**Short description**
AEC	It performs active electrode compensation, as in Brette et al. (2008).
generateCurrentTime	It returns the current simulation time (i.e., from the time the overall Simulink model started).
currentFromConductances	It receives synaptic conductance values and calculates resulting currents to be injected intracellularly.
firingFrequencyClampPID_spikeTriggered	It implements a PI controller, clamping the firing rate of the neuron.
voltageHolder_currentClamp	By a PI controller, it holds (in current-clamp) the membrane potential at a desired value, by regulating the holding current.
G_Poisson_to_Mean_Std	Approximating a Poisson point process synaptic activation by a diffusion approximation (i.e., Ornstein-Uhlenbeck), it computes the process mean and standard deviation.
sin_modulation	It generates sinusoidal signals.
spikeFrequencyMonitor_spikeTriggered	It monitors the firing rate and updates its estimate every time a spike is fired.
timedOutputAndHold	It implements a sample-and-hold block, useful to trigger blocks or output signals changing rarely in time.
WindowedTriggeredSpikeDetector	It detects spikes within a time window.
customIncrementCounter	It counts events.
HH_Euler_step	It implements the Hodgkin–Huxley model of spike initiation.
LIF_simple	It implements the leaky integrate-and-fire model of spike initiation.
QIF_simple	It implements the quadratic integrate-and-fire model of spike initiation.
TraubMilesReducedModel	It implements the reduced Traub-Miles of model spike initiation, as in Ermentrout and Kopell (1998).
aEIF_model	It implements the adaptive exponential integrate-and-fire model of spike initiation.
ChemicalSynapse	It implements a model of chemical synapse, including short-term depression and facilitation, as in Markram–Tsodyks.
OUnoiseGenerator	It generates a realization of an Ornstein–Uhlenbeck stochastic process (i.e., colored Gauss-distributed noise).

We note that six blocks are devoted to implementing highly simplified mathematical models of neuronal excitability (e.g., HH_Euler_step) and synaptic transmission (i.e., ChemicalSynapse). These allow the user to perform tests and “simulated” experiments, enabling a primitive form of debugging and ultimately complementing the use of (hardware) model cells to be connected to the electrophysiological amplifier.

#### Creating new custom blocks

In addition to the basic blocks provided in our Library and the vast amount of general-purpose blocks provided directly by Simulink, new blocks can be created readily, offering unlimited customizability and extensibility. Creation requires a number of steps:

1. Use the “Subsystem” block, provided by Simulink, as a starting template.
2. Instantiate and combine existing blocks, provided by the standard Simulink library or by our Library.
3. If MATLAB code is available as textual scripts, use instead the default MATLAB Function block, while copy-pasting in it the script(s).
4. C code can also be included, use the *ad hoc* blocks S-Function (Builder).
5. As an optional step, add a “mask” to the subsystem, enabling the construction of a GUI-like interface and allowing easy documentation and interface creation.
6. Add the resulting block to the library, as described in Simulink's documentation as well as following our example (i.e., folder NeuroAssets_xPC of the software repository).

The new block is created as a.slx file, including a special file slblocks.m, whose content is:


  function blkStruct = slblocks
  blkStruct.Browser.Library = <<<.slx
file name >>>;
  blkStruct.Browser.Name = <<< library
name >>>;
  end


Having completed these steps, the new block becomes available to use from the default Simulink Library.

#### Demonstration models

In addition to the elementary blocks listed in Table 1, a number of demonstration models is also provided with our Library. These exemplify and take direct advantage of our blocks and of the standard Simulink blocks. A list of these demonstrators (i.e., folder MyModels of the software repository) is provided in Table 2.

**Table 2:** **Demonstrator models provided with the Library**.

**Model folder name**	**Task**
arbitraryStream_and_record	It injects a current, recording the voltage response.
arbitraryStream_and_record_ConductanceClamp	As arbitraryStream_and_record but working with conductance-based injected signals as in dynamic-clamp.
arbitraryStream_and_record_TEST_fclamp	Demo of firing frequency clamping, via PID current feedback.
arbitraryStream_and_record_TEST_synapticInputs	It implements conductance-clamp by simulated synaptic inputs (diffusion approximation).
arbitraryStream_and_record_TEST_vclamp	It provides support for holding the membrane potential by PID regulating the injected current.
EPSP SIZE CLAMP	Demo of clamping the response size, evoked by extracellular stimulation.
RESPONSE PROBABILITY CLAMP	As EPSP SIZE CLAMP, but regulating the probability, evoked by extracellular stimulation.
sine_modulated_conductance_rates_andCurrent_NOSynapticFiltering	It implements temporally modulated conductance injection, upon diffusion approximation.
sine_modulated_conductance_rates_andCurrent_withSynapticFiltering	As the previous but including the low-pass contribution of synaptic filtering.

#### Creating/extending models

Beyond illustrating the use of our Library as a series of case studies, discussed in the next sections, the demonstrators represent a starting point for the creation and extension of the Library. They provide functionalities for (non) conventional cellular electrophysiology experiments and exemplify how simple models/architectures can be used for more sophisticated protocols. All these models are based on arbitraryStream_and_record, offering the essential task required for of recording the neuronal response while simultaneously injecting a stimulus waveform.

Other demonstrators illustrate how to build up on the simple functions of arbitraryStream_and_record and increasingly complex tasks, as closed-loop regulation of membrane potential (i.e., as in voltage-clamp), conductance-clamp, firing rate feedback-control.

The starting point for further customizing and extending our Library is then arbitraryStream_and_record and combine together the elementary blocks of Table 1.

#### Additional MATLAB control and analysis scripts

Together with the actual blocks and demonstrator, we also provide MATLAB scripts describing and implementing the setup of each model parameter, of the running environment, and of other properties on the host PC. These scripts are named following the convention setup<<model_name>>.m and are complemented by a general template, setup_specific_model_TEMPLATE for new blocks. These scripts *de facto* represent an effective means for configuring and quickly launching the models, in an automatic manner, so that a sequence of experimental protocols can be for instance run one after the other from the MATLAB prompt or from a master script, ultimately minimizing dead-times during an experiment.

A set of very basic off-line analysis script is also provided with our Library, e.g., for the estimate of passive membrane properties, for the detection and counting of action potentials, etc. These demonstrate how additional analysis can be easily employed by the user on-the-fly or for off-line data evaluation, from the Host PC.

In the following, we present and discuss the practical use of some of the components of our Library while illustrating a series of sample applications.

### Conventional (open-loop) current-clamp cellular electrophysiology

We first introduce a simple model, obtained by connecting together and configuring the blocks of our Library to implement conventional protocols for *in vitro* neuronal electrophysiology. The execution of this model involves exclusively the *target PC* (see the Materials and Methods and Figure 1) that is interfaced to a patch-clamp amplifier by a DAQ hardware board. The model is outlined in the simplified diagrams of Figure 2, in an attempt at emphasizing the modular nature of our Library, upon combining together its primitives (Figures 2A–C). Each of these blocks performs a specific task and has been often reused throughout the examples. For instance, the Input/Output_DAQ block (Figure 2C) contains all elements necessary for digital-to-analog and analog-to-digital signal conversion and can be easily adapted by the user when DAQ boards other than the one used here are available. The entire Simulink model of Figure 2D records the analog voltage waveform output by the amplifier (i.e., pipette electrode voltage or current) while synthesizing the command voltage signals to the amplifier (e.g., holding voltages or currents, or time-varying current-clamp stimuli). The Input/Output_DAQ block hides the low-level hardware details from the user and exposes directly the pipette electrode voltage and current values, after the application of amplifier-specific conversion unit factors (here indicated in Figure 2C as unitary, solely for the sake of illustration). Specifying or altering these values is thus within immediate reach to the user, upon block properties editing or by launching the MATLAB scripts that we provide in our Library for simplifying its initial installation and configuration.

The ModelParameters block (Figure 2A) provides the current execution time, the solver time-step, and the operation of the active electrode compensation (see below). These parameters (and others in later models) can be read or even modified at run-time by the user, upon interacting with the *host* PC, and are shared among blocks by the graphical representation of virtual wires.

Arbitrary stimulation waveforms, necessary for instance to inject a time-varying current stimulus into the neuronal soma, are next provided via the InputData block (Figure 2B). This supplies data points that are initially specified from a file, generated on the host computer at the MATLAB prompt, or by means of other signal generator software. In our Library, we provide as an add-on the very same waveform-generating engine that was developed and employed in the CLE software package (Linaro et al., 2014). This allows the definition of pulses, ramps, sinusoids and fluctuating waveforms as well as their concatenation and algebraic combination.

**Figure 3:**
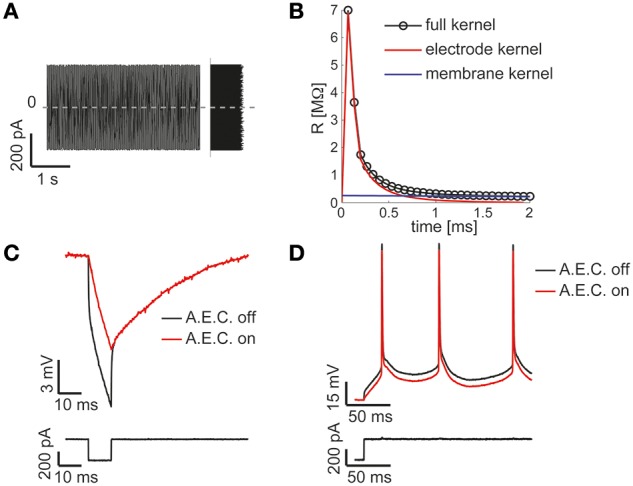
**The Active Electrode Compensation (AEC) is built in and available for any application**. Our Library is one of the very few software platforms available so far to incorporate the AEC by default (Samu et al., 2012; Linaro et al., 2014). Following the method proposed in (Brette et al., 2008), upon intracellular injection of a uniformly distributed fluctuating current **(A)**, the equivalent transfer properties of the patch-pipette electrode are inferred by the corresponding recorded voltage response (not shown) during each experiment. A linear, non-parametric, dynamical model (i.e., the kernel) is identified and distinguished from the passive membrane response components **(B)**. When the AEC block is enabled and graphically connected to the output of the DAQ block (as in Figure 2D), it performs seamless digital subtraction of the instantaneous current injected through the pipette from the recorded voltage, after convolving the current with the estimated electrode kernel. The impact of AEC on subthreshold **(C)** and suprathreshold responses **(D)** of the membrane potential of a neuron is then reminiscent, yet more accurate, of the compensation of pipette resistance and pipette capacitance neutralization, usually performed in hardware by the patch-clamp amplifier.

Combining together the blocks of Figures 2B,C, the waveform data file read from disk represent the current-clamp stimulus injected into the patched cell (see Figures 3C,D, lower black traces). Such current injection is performed with the output data recording (i.e., the pipette electrode potential), implementing the core of all conventional stimulus-response open-loop protocols. In the forthcoming examples, the very same block is used to generate other signal waveforms within distinct models, explicitly demonstrating the reusability of the library components.

**Figure 4:**
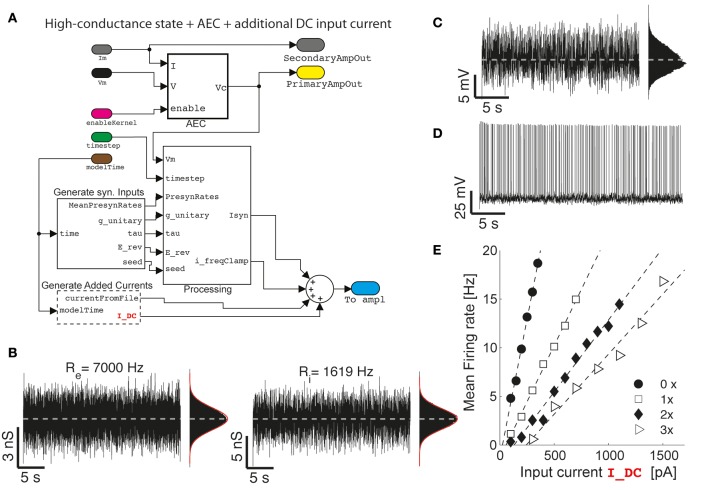
**The impact of artificial background synaptic conductances (stationary) injection**. Upon combining and interconnecting together the blocks of Figure 2 with other two blocks provided in our Library **(A)**, fluctuating excitatory and inhibitory synaptic conductance are generated **(B)** with realistic properties of cortical activity *in vivo*, such as an irregular barrage of events characterized by an equivalent mean presynaptic firing rates (i.e., *R_e_* and *R_i_*) as described earlier (Bal and Destexhe, 2009). As already well-known (Chance et al., 2002; Destexhe et al., 2003), the impact of such an artificial *in vivo*-like synaptic background induce a steady membrane depolarization and random fluctuations in the potential of the neuron **(C)**, irregular firing **(D)**, and increasingly bends the single-cell frequency-current curve reducing its slope (**E**) (i.e., 0×: *R_e_* = *R_i_* = 0 Hz; 1×: *R_e_* = 7000 Hz; 2×: *R_e_* = 14,000 Hz; 3×: *R_e_* = 21,000 Hz; with *R_i_* calculated to “balance” on the average the injected current—i.e., by solving 0 = 〈*G_e_* (*t*)〉 (*E_e_* − *V_b_*) + 〈*G_i_*(*t*)〉 (*E_i_* − *V_b_*) with *V_b_* set 1–2 mV below the voltage firing threshold).

**Figure 5:**
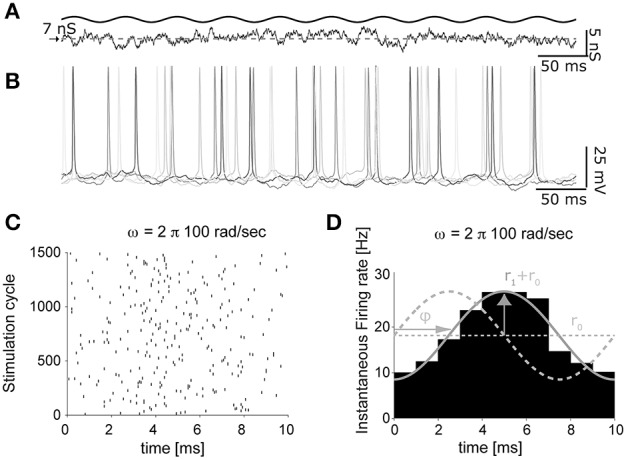
**The impact of artificial background synaptic conductances (non-stationary) injection**. The protocol of Figure 4, was adapted to feature time-varying presynaptic firing rates **(A)**, oscillating sinusoidally in time. The instantaneous firing response, evoked by an input oscillation of 100 cycles/s **(B)**, was then processed to extract the times of each spike, across several repeated trials (i.e., cycles of the background synaptic conductance oscillation), and represented as a raster diagram **(C)**. Once the spike times histogram is evaluated **(D)**, the linear dynamical response of the neuron can be quantified as the average *r*_0_ and the peak firing rates *r*_1_, as well as the phase ϕ with respect to the input cycles.

A key component of the diagram of Figure 2D, is the AEC block and implements the non-parametric Active Electrode Compensation (Brette et al., 2008) of current-clamp recording artifacts in single-electrode intracellular experiments. Indeed, for any application that involves the injection of a current through the same patch-pipette employed for measuring the neuronal membrane voltage response, our Library offers this built-in support. By this technique, the convolution between the (instantaneous) injected current and the impulse response (i.e., kernel) of a linear system, to be experimentally identified to capture the pipette electrode properties, is computed and subtracted in real-time from the potential recorded by the electrode. This method generalizes to a non-parametric and more accurate identification, the conventional bridge-balance and capacitance neutralization techniques usually implemented in hardware. In order to employ the AEC block, the pipette electrode kernel needs first to be estimated, requiring the synthesis and injection of a fluctuating current waveform, with random uniform amplitude distribution. Then the pipette electrode potential (Figure 3A) is recorded, with the amplifier bridge-balance and capacitance compensation circuits disabled and with the AEC block also disabled (Figure 2). As detailed elsewhere (Brette et al., 2008), such a recorded trace allows the identification of the patch pipette electrode kernel (Figure 3B), which is then provided by custom MATLAB scripts, included in our Library, and “uploaded” to the AEC block (see the AEC block documentation or refer to the setup script of the model named setup_arbitraryStream_and_record.m). Once equipped with the patch pipette kernel and enabled, the AEC block can be employed in a variety of applications, such as every other sample experiment reported in this paper. In particular, the use of AEC is strongly recommended for those applications where the *in vivo*-like recreation of background synaptic inputs is achieved by synthetic conductance and not current injection (Figures 4, 5). In fact, in those applications a closed-loop current injection is performed, proportionally depending at every single moment on the measured pipette potential. If the last contains artifactual contributions from the current itself, dynamical instability can rapidly emerge making the conductance injection impossible or compromising the recording stability, upon positive feedback.

The output signals labeled PrimaryAmpOut and SecondaryAmpOut (Figure 2D) are used to log to the memory both the injected current and the membrane voltage (i.e., the compensated pipette electrode potential), at each time-step of the model execution by an additional *ad hoc* block (not shown). The stored data is next transferred for analysis and plotting to the *host* PC, off-line (e.g., by the end of each stimulation trial) by means of the MATLAB scripts provided (see the script setup_arbitraryStream_and_record.m).

The model of Figure 2 can therefore be employed to identify passive and active input-output neuronal membrane properties (i.e., the membrane input resistance and capacitance, the frequency-current curve, etc.) by standard current-clamp protocols. We note that the digital compensation of patch electrode artifacts, enabled by the AEC in our Library, has been made available in very few other real-time software platforms so far (Samu et al., 2012; Linaro et al., 2014).

### (Non) stationary conductance-driven synaptic background recreation

As a second application, we demonstrate how the basic model shown on Figure 2 can be extended and reconfigured (Figure 4A) to perform classic conductance-clamp experiments (Robinson and Kawai, 1993). In these experiments, the activation of somatic ionotropic synaptic receptors, upon (e.g., repeated asynchronous) synaptic release events, is mimicked by a current-clamp stimulus waveform delivered through the patch-pipette. However, the instantaneously injected current amplitude *I* must be continuously adapted to be proportional to the recorded (i.e., AEC compensated) neuronal membrane potential *V* and to the desired instantaneous conductance waveform *g*, thus following the Ohmic relationship *I = G (E - V)*, with *E* a virtual reversal potential. This of course requires a real-time feedback system, since the actual current amplitude needs to be updated at each time (Figures 4, 5), and it is not available on all commercial software/hardware platforms (but see e.g., CED Dynamic Clamp; Benda et al., 2007).

We now first explore a specific case, where a (stationary) barrage of large number of excitatory and inhibitory synaptic inputs irregular activation is recreated. In the details, the time of presynaptic events occurrence are assumed to be highly irregular, with a mean occurrence rate specified as *R_e_* and *R_i_* for the excitatory and inhibitory (virtual) synaptic populations, respectively. In addition, the mean unitary synaptic conductance *g_e_* and *g_i_* are conventionally set to 2 and 6% of the resting input membrane conductance of the neuron (Chance et al., 2002), and the apparent reversal potentials are set to *E_e_* = 0 *mV* and *E*_i_ = −80 *mV*, for the excitatory and inhibitory synaptic currents, respectively. These inputs and their parameters are seamlessly integrated into the model using the GenerateSynapticInput block (Figure 4A). The resulting injected current can thus be represented as the sum of two components (i.e., generated by the custom block Processing):

(1) $$I_{syn}(t)=G_{e}(t)\left(E_{e}-V(t)\right)+G_{i}(t)\left(E_{i}-V(t)\right)$$

Under the hypothesis of a linear summation of individual contributions, and given the rather small resulting amplitude of the resulting single postsynaptic currents, the specific details of synaptic receptor kinetics can be specified equivalently, under the diffusion approximation (Bal and Destexhe, 2009), in terms of the autocorrelation time-length of a fluctuating conductance waveform. This has been chosen as *τ_e_* = 5*ms* and *τ_i_* = 10 *ms* for the excitatory and inhibitory components, respectively. The fluctuating waveforms are then internally generated on-line, as realizations of Ornstein–Uhlenbeck processes, by numerically integrating two independent stochastic differential equations (Uhlenbeck and Ornstein, 1930):

(2) $$\frac{d}{dt}G_{x}=\left({\bar{G}}_{x}-G_{x}\right)/\tau_{x}+\sqrt{2D_{x}}\;\xi_{x}(t),$$

with *x* ∈ {*e, i*}, and where *ξ_x_*(*t*) is an independent realization of a zero-mean Gaussian-distributed white noise, with unitary variance. This is performed for both the excitatory and inhibitory conductance *G_e_* and *G_i_* by the OU block (not shown), which incorporates fast C/C++ code. For each of the two equations, the mean *G_x_* and standard deviation *G*^2^_*Sx*_ are related to the virtual presynaptic activation rates, as (Bal and Destexhe, 2009):

(3) $${\bar{G}}_{x}=g_{x}\;\tau_{x}\;R_{x}\;G_{Sx}^{2}=\frac{1}{2}g_{x}^{2}\;\tau_{x}^{2}\;R_{x}/\tau$$

with *x* ∈ {*e, i*}. These values are generated by the block G_Poisson_to_Mean_Std, within the subsystem Processing—Figure 4B, requiring no programming from the user.

For a choice of realistic stimulation parameter (see the caption of Figure 4) which approximate the so-called high-conductance state (Destexhe et al., 2001; Steriade, 2001; Litwin-Kumar et al., 2011), an overall mean depolarization of the membrane of 10–15 mV and fluctuations of ~5 mV (Figure 4C) occur and it accompanied by an irregular emission of action potentials (Figure 4D). The last is accompanied by a distribution of inter-spike intervals with an approximately exponential distribution (Chow and White, 1996; Ostojic, 2011).

While this regime of neuronal activity has been suggested to better approximate some of the operating conditions neurons experience in the intact cortex, it represents a simple way to reintroduce artificially some degree of realism in cellular electrophysiology experiments carried out in acute brain slices, which largely miss any spontaneous background activity.

However, the impact of stationary background recreated synaptic activity leads to other known consequences, relevant for understanding the single-cell integrative properties. If an additional (e.g., constant-amplitude) external current *I_ext_* component is added and specified from a file (as in Figure 2B) or generated at run-time by the GenerateAddedCurrents block (Figure 4A), a modulation of the slope of the frequency-current (F-*I_ext_*) relationship of neurons can be revealed upon altering the background activity. To this end, excitatory input rates were set to increasing values *R_e_* ϵ {0, 7000, 14,000, 21,000} Hz, and *R_i_* calculated appropriately (see the caption of Figure 4), to balance the average (recreated) synaptic current near the firing threshold of the neuron. Under these conditions, the slope of the F-*I_ext_* decreases with increasing intensity of background conductance as in Chance et al. (2002).

The same model depicted in Figure 4A can be immediately adapted replacing the external constant amplitude current *I_ext_* by a temporally modulated waveform, exploiting the GenerateAddedCurrents block. This involves the direct generalization of the experimental conditions first employed in Köndgen et al. (2008) and Brunel et al. (2001). An even more interesting generalization of those experiments, to the case of time-varying recreated background synaptic activity, is represented by the temporal modulation of the mean activation rates of the virtual presynaptic population of excitatory and inhibitory neurons (Figure 5A), described at the beginning of this section. By using the block sin_modulate_rates and including it in the model of Figure 4A, at each time-step of the model execution, the background synaptic activation rates can be made sinusoidally modulated in time:

(4) $$R_{x}(t)=R_{x0}+R_{x1}\;sin(\omega \;t)$$

with *x* ∈ {*e, i*}, *R*_*e*0_, *R*_*i*0_, and *R*_*e*1_, *R*_*i*1_ representing the offset and peak amplitude for each of the conductance components, and where ω is the modulation frequency (i.e., in radiant) and *t* is the model time (Figure 1A).

As documented in Figures 5B–D, the time-varying firing probability of cortical neurons can be studied as a function of the time-varying (recreated) synaptic inputs, identifying simple dynamical linear transfer properties, such as magnitude and phase of the neuronal response (Figure 5D) (Köndgen et al., 2008; Testa-Silva et al., 2014).

In these sample experiments, the results of which will be published, presented and detailed elsewhere (see also Biro et al., 2013), the peak firing rate amplitude (*R*_x1_) was 10% of the offset value (*R*_x0_) and could be added to the excitatory inputs, the inhibitory inputs, or both components simultaneously. Neuronal firing responses to such temporally-modulated background synaptic inputs repeated for many trials (Figure 5B) were analyzed in terms of the occurrence of individual action potentials (Figure 5C), and displayed with respect to the cycle of the input stimulation (Figure 5D) by the peristimulus time-histogram. Using this method, the instantaneous rate *r*(*t*) of output firing of the patched neuron could be best fit by a sinusoid:

(5) $$r(t)=r_{0}+r_{1}\cdot sin\left(\omega \;t+\varphi \right)$$

Where the response magnitude and phase were expressed as *r*_1_ and ϕ.

**Figure 6:**
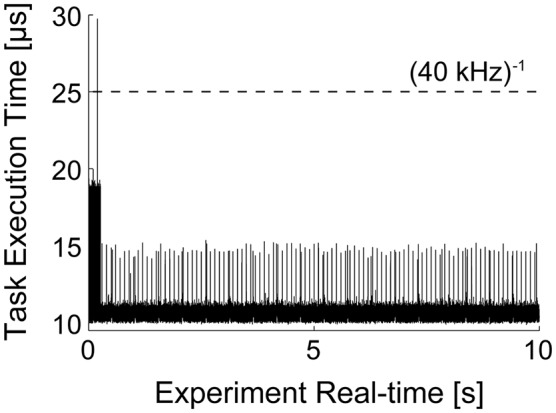
**Task execution time (TET) of model presented in Figure 5**. The MATLAB/Simulink-xPC combination allows the monitoring of performance of the target PC, while executing the specified model. This makes possible to assess whether the computational loads, imposed e.g., by the recreated *in vivo*-like background synaptic activity and by the PID automatic regulation of the neuronal firing frequency, are compatible with the model operation in real-time. The initial overhead and the periodic peaks are a consequence of the firing-rate (see Section Clamping the Neuronal Firing Rate and **Figure 7**) and of transient loads of non-optimized routines. Nonetheless, the model could be run at sampling frequencies higher than 50 kHz.

It is noteworthy to illustrate the performance of the real-time system, under the conditions of Figure 5. Under moderate protocol complexity, involving acquiring external input waveforms from file, continuous logging of output data to the memory, handling analog inputs and outputs via a DAQ board and, at the same time updating time-varying parameters of the background synaptic activity for both the excitatory and inhibitory components, the model could be run on the *target* PC at a high sampling rate (i.e., 15–20 kHz). When the Task Execution Time was explicitly monitored, indicating the time taken by each iteration of the real-time loop [i.e., iteration of Equation (2) and management of analog I/O], a direct insight on the maximal system performance could be obtained (Figure 6). One iteration of the model took at least 10 μs (i.e., corresponding 100 kHz) and at most ~30 μs (i.e., 33 kHz). Indeed, this model could be operated at rates of up to 50 kHz without any error and further fine-tuning can be easily made to the block provided in our Library, in case faster execution rates are necessary. Whenever hard-real time is strictly required, or when a guarantee on model executing time is needed, the Simulink/xPC can be instructed to stop the execution of the model and return an error. In our experience, this allowed us to perform optimization of the blocks and to gain more insight on the maximal hardware performance allowed when running a specific model.

### Clamping the neuronal firing rate

**Figure 7:**
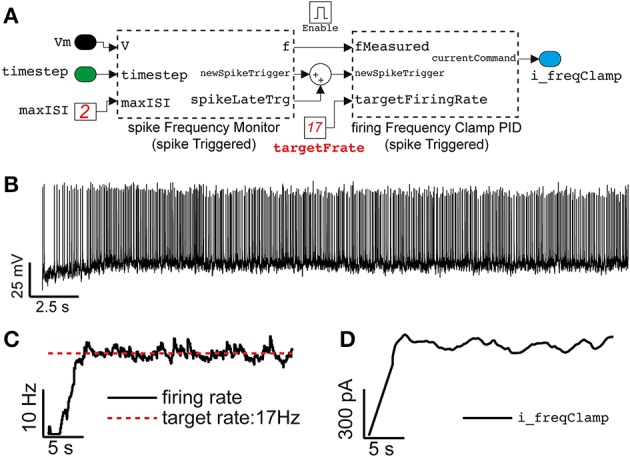
**Close-loop control of the neuronal mean firing rate, in the same model of Figures 4, 5**. Adopting the approach outlined in Miranda-Dominguez et al. (2010), Linaro et al. (2014), an elementary Proportional-Integrative-Derivative (PID) controller **(A)** is demonstrated to automatically tune the value of the external current input current (see Figure 4A) to (initially) constrain the average neuronal firing frequency to a desired value **(B,C)**. Despite the irregular firing (recreated) regime (i.e., *R_e_* = 7000 Hz, *R_i_* = 2149 Hz), the PID controller adapts in real-time *I_ext_* **(D)** on the basis of a feedback reaction (i.e., *P* = 0.0045, *I* = 0.0023, *D* = 0, see the Results).

An additional component useful for the models employed in Figures 4, 5 can be reused and employed to automatically adjust stimulation parameters and quickly have neuronal firing rates approach a desired range (Miranda-Dominguez et al., 2010; Linaro et al., 2014). This has been particularly useful to minimize the average duration of an experiment while adjusting the amplitude of an external current *I_ext_* to reach a similar output-firing regime across many distinct experiments. Figure 7 reports on the use of this technique, simultaneously to the conductance-clamp paradigms that we have discussed already. The ClampFiringFrequency block (Figure 7A), provided by our library, implements this protocol and contains two custom components: the first (spikeFrequencyMonitor_spikeTriggered) contains custom MATLAB code to monitor the spike rate of the neuron upon a sliding averaging window. It updates its output at the arrival of each new action potential and instantaneously reports a weighted average between the previous firing rate history and the inverse of the last inter-spike interval.

The second component (firingFrequencyClampPID_spikeTriggered) receives the estimated firing rate and implements a Proportional-Integrative-Derivative (PID) controller to adjust and regulate in real-time the value of the feedback external current required to make the neuron fire approximately at the desired rate. This block also updates its own output only at the arrival of a new action potential, as in a sample-and-hold system where the most recent external current amplitude value is kept fixed until the next update.

A representative case is depicted in Figures 7B–D, illustrating how the average firing rate of a neuron could be clamped to ~17 Hz, using this approach, while also receiving stationary (recreated) background fluctuating inputs.

### Response clamp, upon extracellular stimulation

A further generalization of the closed-loop protocol, presented in the previous section and illustrated in Figure 7, includes the combination of intracellular and extracellular electrophysiological methods for eliciting and recording synaptic and action potentials upon extracellular electrical stimulation. Here we demonstrate and discuss how the probability of evoking an action potential as well as the efficacy amplitude of an elicited compound postsynaptic potentials (EPSPs) can be controlled in closed-loop, upon automated regulation of the intensity of an extracellular electrical stimulus, via a PID. In our experimental setup (Figure 1), combining intracellular and extracellular techniques required the coordination of a hardware stimulus isolator (see the Materials and Methods and Figure 1) and of the intracellular access to the membrane potential (as in Figure 7), while providing real-time access and analysis of supra and sub-threshold membrane voltage dynamics.

The extracellular stimuli were applied to the acute cortical slice, via a substrate-integrated microelectrode array (MEA—see the Materials and Methods), which replaced the bottom of the chamber employed for patch-clamp. Out of the 60 available, a pair of microelectrodes located (i) in proximity of the soma (L5) of the patched cell or (ii) of its apical dendrites (L1) was selected. Under pharmacological blockade of synaptic transmission, the first case allows one to study antidromic action potential responses (or their failures), while under control conditions the second case allows one to study compound postsynaptic currents.

**Figure 8:**
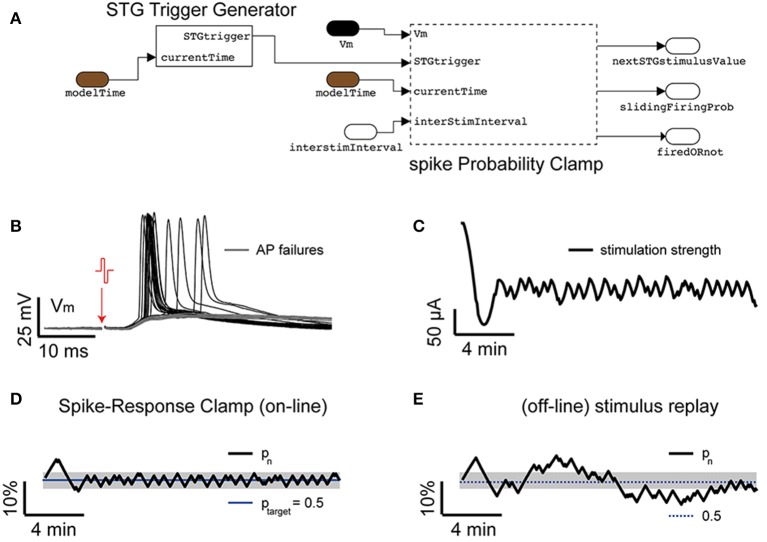
**Response clamp**. *Ad hoc* blocks are also available in our Library **(A)**, performing refined closed-loop protocols. As first demonstrated in dissociated cell cultures (Wallach and Marom, 2012; Wallach, 2013) the closed-loop control of the spike-response probability, upon extracellular antidromic electrical stimulation **(B)**, provides precious insights on the time-scales of neuronal excitability. By combining the block STGTriggerGenerator and the PID block spikeProbabilityClamp to the protocol of Figure 2, the intensity of a repeated extracellular current-controlled electrical stimulation is automatically regulated in real-time **(C)** to maintain the probability of eliciting an action potential to a desired value **(D)**. However, as soon as the sequence of commands is replayed in open loop **(E)**, the response probability displays larger fluctuations. Commonly used values were *P_pid_* = 1, *I_pid_* = 0.3, *G* = 700.

Focusing first on the case (i), under the pharmacological blockade of synaptic transmission via a cocktail of selective synaptic receptor antagonists (i.e., AP-V, CNQX, and GABAzine) (Reinartz et al., 2014), the model sketched in Figure 8 was assembled by the blocks provided in our Library and employed in combination to the model of Figure 2, to demonstrate feedback regulation of extracellular stimulation intensity as first investigated in Wallach and Marom (2012) and Wallach (2013). The resulting model allows carrying patch-clamp recordings, as in Figure 3, and additionally employs a sliding window estimator of the instantaneous response probability (spikeProbabilityClamp). The Input/Output_DAQ block however now contains an additional signal: the digital trigger, running from the *target* PC to the extracellular stimulator (see Materials and Methods) and is required to launch the extracellular stimulation at the right moment (i.e., inter-stimulus interval of 3–5 s), and elicit an antidromic action potential. This is implemented by the block STGTriggerGenerator (Figure 7A). The membrane voltage is not continuously logged to the memory, but instead it is recorded 10 ms preceding and 500 ms following the delivery of the stimulus (i.e., triggered logging—Figure 8B). This is implemented by the block recordTriggerGenerator (not shown in Figure 7A).

The main closed-loop regulation task is then performed by spikeProbabilityClamp: it detects action potentials upon membrane potential threshold crossing (i.e., by the spikeDetector internal block—not shown) and decides the appropriate intensity of the forthcoming extracellular stimuli (see Equations 7, 8). Both those blocks contain custom MATLAB code, implementing a sliding estimate of the evoked response probability (*p_n_*) at a *n*th stimulus in a sequence:

(6) $$p_{n}=p_{n-1}\cdot e^{-ISI/\tau }+F\cdot \left(1-e^{-ISI/\tau }\right),$$

where *p*_*n*−1_ represents the firing probability value before the current stimulus and *ISI* is the inter-stimulation interval, τ = 600 s and *F* = 1 if an action potential that occurred in the recording time window (i.e., otherwise it is 0). spikeProbabilityClamp next determines the amplitude of the subsequent extracellular stimuli (*S*_*n*+1_) via a PI controller:

(7) $$S_{n+1}=S_{1}+G\cdot \left(P_{pid}\cdot EP_{n}+I_{pid}\cdot EI_{n}\right),$$

with

(8) $$\begin{array}{l} EP_{n}\;=\;p_{target}\;-\;p_{n}\\ EI_{n}\;=\;EI_{n-1}\;+\;EP_{n} \end{array}$$

In the above equation, *P_pid_* and *I_pid_* are the corresponding proportional and integral-control coefficients, and *n* denotes the current iteration cycle (i.e., the current stimulus number). *G* represents the gain and *S*_1_ a constant offset, specified as an initial value of the stimulation sequence. Such a feedback will achieve after just few iterations, the targeted response probability (*p_target_*).

Figure 8D reports an example of response clamp, where the PID regulation achieved very good control of the response probability, with a tolerance lower than 10%. The relevance of examining the sequence of stimulation amplitudes (Figure 8C) required to clamp the response probability carries, as discussed, extremely rich information on the underlying neuronal effective excitability and a similar explicative power as the voltage-clamp had in the dissection of the ionic basis of the action potential initiation (Wallach and Marom, 2012; Wallach, 2013). As in the earlier experiments carried out on dissociated cell cultures, replaying the very same sequence of stimuli again (Figure 8E) leads to a different evolution of the response probability, whose variance is higher than in closed-loop, and reveals an ever changing temporal evolution of the intrinsic effective excitability of the neuron, which depends on the variability of the response itself and cannot be observed directly in open-loop.

Besides the multiple intrinsic factors determining the diverse time-scales of neuronal excitability, the response probability also depends on the value of the membrane potential value at the moment of stimulus arrival. By the intracellular access to the membrane potential, it may therefore be desirable to constrain the membrane voltage to a repeatable desired, sub-threshold value. Such a variant to the original protocol of the response-clamp by Wallach and Marom, can be achieved by our Library, employing the voltageHolder_currentClamp block. This adds to the scheme of Figure 8A, an additional PI controller (e.g., *P_pid_* = 10, *I_pid_* = 100) to automatically constrain the membrane potential to the same value (Figure 8B) upon an automatically adapted external (holding) current. An additional important feature offered by this block is the option to switch from active (PI) control to an open-loop holding of the last external injected current, with the aim of avoiding any interference with the responses elicited by the extracellular stimuli. Indeed, this additional PI controller can be instructed to be temporarily disconnected from its input, from 10 ms before the stimulus arrival until 500 ms after, resulting in no update of its output.

**Figure 9:**
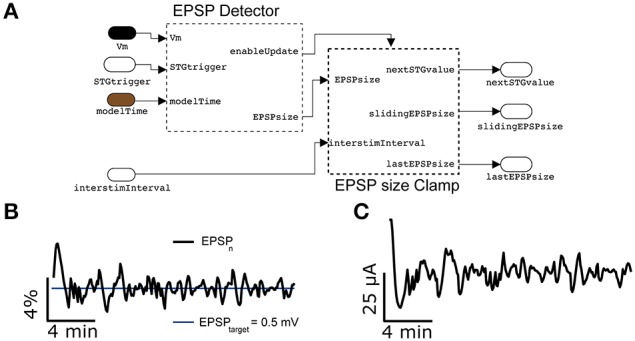
**Synaptic response clamp**. Further generalizing the experimental protocol of Figure 7 to electrically evoked synaptic responses, measured postsynaptically by an intracellular access to the neuron, the intensity of the peak compound evoked Postsynaptic Potential (PSP) can be automatically regulated by a PI controller **(A)**, provided in our Library. In this case, thanks to the EPSPsizeClamp module, the evoked PSP peak amplitude can be constrained to a desired value of 0.5 mV with a tolerance of less than 4% **(B)**, while adapting in real time the intensity of the extracellular stimulation **(C)**. Commonly used parameter values were *P_pid_* = 1, *I_pid_* = 0.3, *G* = 1′600, τ = 300 s.

Replacing the spikeProbabilityClamp subsystem on Figure 8A by the EPSPclamp block (Figure 9A) under normal recording conditions and electrically stimulating in L1, makes it possible to clamp the efficacy of evoked postsynaptic potentials (PSPs) and explore its consequences, as done for the response probability (Figure 9B) (Reinartz et al., 2014). This subsystem contains a customized block to detect the PSP amplitudes evoked by extracellular stimulation. A sliding temporal average of the PSP amplitude is then calculated internally as in Equation (6), replacing the variable *F* with the peak amplitude of the last elicited PSP. As in response clamp, the feedback control of the stimulator intensity is computed as in Equation (8), where the PSP target amplitude replaces the target response probability.

Figure 9B reports a representative example, where the amplitude of compound PSPs elicited by L1 extracellular stimulation was clamped to 0.5 mV with an accuracy of less than 4%.

Such (synaptic) response-probing methods (Figures 8, 9) have proven very useful for gaining insight into neuronal excitability and synaptic efficacy, as reported elsewhere (Reinartz et al., 2014).

## Compatibility and interfacing

The compatibility and interfacing of our Library, in terms of models, block, and overall platform, with other software toolboxes or hardware equipment is constrained by the overall compatibility of these toolboxes and hardware to MATLAB and Simulink. There are several options available.

### Integration of existing code

As pointed out previously, blocks can be built from scratch or customized as user-additions to the Simulink library. Thanks to support of Simulink for C and MATLAB code, any function/script/code of third parties software can be included into a *ad hoc* Simulink block. This requires only minor modifications of the original code and allows compatibility and interfacing by embedding the desired functions into Simulink. As such, real-time aspects are also fully preserved.

Using communication protocols and control signals. Should third parties software run on a different PC, or in case when interaction with dedicated hardware is necessary (e.g., a stimulus generator, microcontrollers, etc.), TCP/IP, UDP (Ethernet), or serial (i.e., RS232, USB) communication protocols can be used. By default, Simulink offers specific blocks that handle such communication. We however note that real-time operation of such communication protocols cannot be guaranteed, limiting the applicability in cases where very precise timing requirements are not relevant. Nonetheless, as analog and digital input/output can be interfaced or exchanged with the *target* PC by its AD/DA hardware, TTL triggering can be employed if externally supported, offering real-time communication.

### Using the host PC

*Host* and *target* PCs communicate via TCP/IP, particularly during model initialization and execution. Such communication can be exploited employing the *host* PC as an intermediate layer of communication: e.g., the *host* PC monitors (under MATLAB) the state of the xPC model running on the *target* PC by commands tg.getsignalidsfromlabel and tg.getsignal, tg being the internal MATLAB reference to the target. Issuing appropriate (system) calls to the external software/hardware under MATLAB on the *host* PC, in response to monitored responses or change in status of the xPC target, would implement the interfacing. This is useful with system calls, invoking Dynamic-link libraries (DLL) or executing MATLAB code on the *host* PC, despite intrinsic limitation in real timing precision imposed by the Ethernet communication jitter.

In Section Response Clamp, Upon Extracellular Stimulation (see also the Materials and Methods), we gave an example of such an interfacing: the real-time model running on the *target* PC monitors the intracellular voltage response evoked by a repeated extracellular electrical stimulus. It then calculates the intensity of the next stimulus according to a (PID) control algorithm. It is then the *host* PC that periodically reads the next stimulus intensity (by tg.getsignalidsfromlabel and tg.getsignal) and (re)configures an external stimulus isolator, connected by USB, by a DLL call. The real-time coordination was guaranteed by TTL triggering the stimulus isolator by a digital output generated by the xPC model. A similar combination of interfacing strategies allowed us to avoid time-consuming USB communication on the *target* PC while ensuring task coordination.

Interfacing our Library to other hardware and software tools (running on the *host* PC or on a different computer) can therefore take place as for the case of the stimulus isolator, and including tools such as Psychtoolbox (Brainard, 1997), VideoToolbox (Pelli, 1997), brain computer interfaces (Delorme et al., 2010 and references therein), the Biological Neural Networks Toolbox (http://www.ymer.org), NeuroRighter (Rolston et al., 2010; Newman et al., 2013), RTXI (Lin et al., 2010), LCG (Linaro et al., 2014), and other software. In all cases, while digital triggering provides real-time synchronization, non-real-time communication is possible via the *host* PC.

## Conclusion

In this contribution we introduced and validated our Library of Simulink custom blocks, over a series of common experimental protocols involving real-time closed-loop operation. A major feature is the native implementation of the Active Electrode Compensation technique (Brette et al., 2008), as well as the possibility to perform fluctuating conductance-waveform injections (Bal and Destexhe, 2009) and its extension to non-stationary regimes (Köndgen et al., 2008).

Ultimately, the strength of our approach is that the Library is built on the extensive support, by the real-time operating system, of a variety of hardware including DAQ cards from many vendors and standard serial ports. As demonstrated in Figures 8, 9, slower devices can also be interfaced (e.g., via USB) on the *host* PC, extending the possibilities and compatible equipment. In our case, this involved a multichannel extracellular stimulator for the spatially patterned delivery of electrical pulses through substrate-integrated microelectrode arrays, combined and synchronized with patch-clamp recordings. Additional closed-loop protocols could then be implemented, probing non-trivial neuronal and synaptic properties such as those enabled by the firing response probability (Wallach and Marom, 2012; Wallach, 2013) or upon the control of compound postsynaptic potential amplitude (Reinartz et al., 2014).

Despite a number of pre-built models and blocks provided, useful for quick deployment of basic experimental demands including real-time tasks, our Library should be regarded as a starting point for further developments and customizations. We are convinced that a strength of this approach compared to others (Benda et al., 2007; Lin et al., 2010; Rolston et al., 2010; Newman et al., 2013; Linaro et al., 2014) is the simplicity of use and customization, building on the advantages of a visual programming environment where the available default blocks can be assembled together in a LEGO-like fashion. Importantly, the ease of extension is supported not only by the default Simulink system library, but also through programmatic integration of arbitrary C/C++/MATLAB code into new blocks, as we explicitly benefitted from in our Library. In conclusion, we believe that the increasing number of MATLAB/Simulink users in research institutions might offer a good environment for novel users and developers of our Library and bring its use to the hands of the experimentalists.

## Information sharing statement

The Library and the MATLAB scripts described here are freely available (https://bitbucket.org/mgiugliano/pc_neuron_simulink) and will be made available from the software repository of the International Neuroinformatics Coordinating Facility.

## Author contributions

This work was carried out in close collaboration between all co-authors. IB and MG defined the research theme and both contributed to the software architecture. IB and MG wrote the manuscript. All authors have contributed to, seen and approved the final version of the manuscript.

### Conflict of interest statement

The authors declare that the research was conducted in the absence of any commercial or financial relationships that could be construed as a potential conflict of interest.
